# Immunomodulatory Mechanisms of Mesenchymal Stromal Cells: Cytokine Networks and Therapeutic Potential Across Immune-Mediated, Inflammatory, and Regenerative Disorders

**DOI:** 10.3390/biology15100794

**Published:** 2026-05-16

**Authors:** Tamerlan Nurlybek, Nursulu Altaeva, Baglan Kazhiyakhmetova, Zhansaya Seitkumarova, Yerkezhan Baidildina, Anastassiya Vizigina, Yerlan Kashkinbayev

**Affiliations:** 1 Department of Medical Genetics and Molecular Biology NJSC, Astana Medical University, Astana 010000, Kazakhstan; nrlbktamerlan@gmail.com (T.N.); kazhiyakhmetova.r@amu.kz (B.K.); rewdkeelul@gmail.com (Z.S.); yerkezhanbai@gmail.com (Y.B.); nastiavizigina@gmail.com (A.V.); 2 Scientific Research Institute of Radiobiology and Radiation Protection NJSC, Astana Medical University, Astana 010000, Kazakhstan

**Keywords:** mesenchymal stromal cells, cytokine networks, immunomodulation, regenerative medicine, autoimmune disease, mesenchymal stromal cell-derived extracellular vesicles

## Abstract

Mesenchymal stromal cells (MSCs) are multipotent cells characterized by their regenerative capacity and strong immunomodulatory properties. In recent years, MSC-based therapy has attracted significant attention as a potential treatment for a wide range of immune-mediated and degenerative diseases. The therapeutic effects of MSCs are primarily mediated through paracrine signaling and secretion of cytokines that regulate immune responses and promote tissue repair. This review focuses on five key cytokines involved in MSC immunomodulation: interleukin-6 (IL-6), interleukin-10 (IL-10), transforming growth factor-beta (TGF-β), tumor necrosis factor-alpha (TNF-α), and interleukin-1 beta (IL-1β). These cytokines interact within a complex signaling network that allows MSCs to suppress excessive inflammation and restore immune balance. The role of MSC therapy is examined in several clinically relevant conditions, including systemic lupus erythematosus, systemic sclerosis, ischemic stroke, spinal cord injury, diabetes mellitus, and female infertility. Across these diseases, MSCs demonstrate the ability to inhibit pro-inflammatory immune cell activity, promote regulatory immune phenotypes, reduce oxidative stress, and stimulate regeneration through the secretion of growth factors and extracellular vesicles. Despite promising experimental and early clinical findings, several limitations remain, including variability in MSC sources, limited cell survival after transplantation, and the need for optimized dosing strategies. Overall, MSC therapy represents a multifunctional therapeutic approach combining immunomodulation, anti-inflammatory activity, and regenerative support. Further research is required to better understand cytokine interactions, improve standardization of MSC-based treatments, and enhance clinical efficacy across diverse pathological conditions.

## Simple Summary

Mesenchymal stromal cells (MSCs) are special cells capable of reducing inflammation and supporting tissue repair. Scientists are studying MSC therapy as a possible treatment for diseases caused by immune system dysfunction and tissue damage. MSCs release important signaling molecules called cytokines, which help control immune responses and prevent excessive inflammation. Key cytokines involved in MSC therapy include IL-6, IL-10, TGF-β, TNF-α, and IL-1β. Research shows that MSCs may help treat conditions such as systemic lupus erythematosus, systemic sclerosis, ischemic stroke, spinal cord injury, diabetes mellitus, and female infertility. MSCs can slow harmful immune reactions, promote protective immune cells, support regeneration of damaged tissues, and improve healing processes. Although MSC therapy is still being actively studied, current findings suggest that it may become an important future treatment for complex diseases involving inflammation and immune imbalance.

## 1. Introduction

Mesenchymal stromal cells (MSCs) are non-hematopoietic fibroblast-like cells that reveal self-renewal abilities and multipotent differentiation potential. The cells now known as MSCs were first described by Friedenstein and colleagues in 1970 as plastic-adherent, fibroblast-like, colony-forming units derived from bone marrow stroma. The term “mesenchymal stem cells” was introduced two decades later by Caplan (1991) [1] to describe their multipotent differentiation potential into chondrocytes, osteoblasts, adipocytes, and myogenic tissue. The International Society for Cell & Gene Therapy (ISCT^®^) currently recommends the term ‘stromal’ over ‘stem’ to more accurately reflect their biological properties [2]. By convention, MSCs are defined by three minimum criteria: plastic adherence under standard culture conditions; expression of surface markers CD105, CD73, and CD90 with the absence of CD45, CD34, CD14, and HLA-DR; and demonstrated capacity for trilineage differentiation in vitro [2].

Beyond their regenerative properties, MSCs have attracted considerable clinical interest due to their potent immunomodulatory capacity and, crucially, their immunoprivileged status. MSCs constitutively lack MHC class II molecules and do not express the co-stimulatory molecules CD40, CD80, and CD86 required for T helper cell activation, rendering them effectively invisible to adaptive immune surveillance and resistant to allogeneic rejection [3]. This immune privilege is further reinforced by the active secretion of anti-inflammatory mediators, including IL-6 and TGF-β1, which collectively suppress effector T cell responses and promote tolerogenic conditions in the local microenvironment [4].

It is important to note, however, that the immunomodulatory role of MSCs is not straightforwardly suppressive. Following systemic intravenous administration, MSCs become physically trapped in the pulmonary microvasculature due to their size, where they trigger an immediate innate inflammatory response characterized by elevated levels of macrophages, neutrophils, TNF-α, IL-1β, and MCP-1 [5]. Despite occurring primarily in the lungs, the inflammatory signals generated propagate systemically via the circulation, reaching secondary organs, including the liver. Only subsequently does the immunosuppressive phase emerge, driven by the secretion of soluble factors including IL-6, IL-10, and TGF-β [6]. The net functional outcome of MSC administration is, nonetheless, immunosuppressive: challenge with lipopolysaccharide (LPS) three days following MSC infusion results in significantly attenuated levels of IL-6 and other pro-inflammatory cytokines compared to control animals, an effect attributable to the transition of macrophages from the pro-inflammatory M1 to the anti-inflammatory M2 phenotype facilitated by MSC-derived IL-10 [5]. M2 macrophages subsequently modulate NK cell activity, suppress CD8+ cytotoxic T lymphocytes, and promote the expansion of regulatory T cells (Tregs), thereby establishing a sustained immunosuppressive environment [6].

A critical practical consideration in MSC-based therapy is the issue of dosing. Administration of 5 × 10^6^ cells/kg has been established as a well-tolerated dose, while a tenfold increase results in 20% mortality in rodent models within 24–48 h of infusion, attributed to the development of thromboembolic complications and disseminated intravascular coagulation (DIC)—a life-threatening coagulopathy characterized by widespread microvascular thrombosis and depletion of coagulation factors [7]. These findings underscore the necessity of precise dose optimization prior to clinical translation.

The immunomodulatory capacity of MSCs is mediated by a network of five key cytokine factors: IL-6, IL-10, TGF-β, TNF-α, and IL-1β. Each of these factors plays a distinct and context-dependent role in the regulation of innate and adaptive immune responses following MSC transplantation, and together, they constitute the molecular basis of MSC-mediated immunosuppression. This review examines the specific immunological effects of each cytokine factor and evaluates the therapeutic potential of MSC-based interventions across six clinically relevant conditions: systemic sclerosis, systemic lupus erythematosus, ischemic stroke and its sequelae, spinal cord injury, female infertility, and diabetes mellitus.

Throughout this review, the term “cytokine network” refers to the integrated, feedback-regulated signaling architecture surrounding MSC immunomodulation, rather than a simple list of molecules. In this framework, inflammatory licensing cytokines (TNF-α, IL-1β, and IFN-γ) derived from the local microenvironment activate MSCs through NF-κB and JAK/STAT pathways; licensed MSCs in turn secrete a coordinated set of effector cytokines and mediators (IL-10, TGF-β, IL-6, IDO, PGE2, and soluble PD-L1); and these effectors generate negative-feedback loops, including IL-10-mediated M1→M2 macrophage polarization that reduces local TNF-α and dampens the original licensing input. The five cytokines emphasized in Section 2 (IL-6, IL-10, TGF-β, TNF-α, and IL-1β) are the most extensively studied nodes within this network and are framed accordingly.

The six conditions selected for this review—systemic lupus erythematosus, systemic sclerosis, ischemic stroke, spinal cord injury, diabetes mellitus, and female infertility—span autoimmune, metabolic, acute neurological, and reproductive categories. They share three pathological features: (i) chronic or acute immune dysregulation, (ii) sustained inflammatory cytokine signaling, and (iii) impaired endogenous tissue regeneration. These three features are precisely the targets of MSC paracrine activity, justifying their joint consideration within a single mechanistic framework.

### Literature Search Strategy

Relevant publications were retrieved from PubMed, Scopus, and Web of Science between January 2000 and October 2025. The search combined the terms (“mesenchymal stromal cells” OR “mesenchymal stem cells” OR “MSC”) AND (“cytokine” OR “IL-6” OR “IL-10” OR “TGF-β” OR “TNF-α” OR “IL-1β”) AND (“systemic lupus erythematosus” OR “systemic sclerosis” OR “ischemic stroke” OR “spinal cord injury” OR “diabetes mellitus” OR “infertility”). Inclusion criteria were peer-reviewed original research, systematic reviews, and clinical trial reports in English. Exclusion criteria were case reports, non-peer-reviewed preprints, and articles without accessible full text. Reference lists of retrieved articles were screened for additional sources. As this is a narrative review, a full PRISMA flow diagram was not generated.

## 2. Cytokine Factors

Mesenchymal stromal cells (MSCs) exert their immunomodulatory effects through a coordinated network of cytokines rather than through isolated signaling molecules. In response to inflammatory cues in their microenvironment, MSCs dynamically regulate cytokine production, thereby orchestrating both pro-inflammatory and anti-inflammatory pathways. This context-dependent cytokine modulation enables MSCs to fine-tune immune responses, suppress excessive inflammation, and promote tissue repair. These cytokines can be broadly categorized into pro- and anti-inflammatory mediators, as well as licensing signals that activate MSC immunoregulatory functions [8].

Table 1 summarizes the principal cytokines discussed in this section, classifying each as an inflammatory licensing input, an MSC-derived effector mediator, or a context-dependent factor.

### 2.1. IL-6

IL-6 is a pleiotropic cytokine with both pro-inflammatory and anti-inflammatory properties and represents one of the earliest mediators elevated following MSC administration [5]. Compared to other cytokines produced by MSCs, IL-6 is secreted continuously, with further upregulation in the presence of IL-1α [4]. Functionally, MSC-derived IL-6 inhibits dendritic cell maturation, impairing their antigen-presenting capacity and subsequent T cell activation [4].

In addition, IL-6 contributes to the regulation of leukocyte trafficking by downregulating adhesion molecule expression on endothelial cells, thereby limiting immune cell recruitment to sites of tissue injury [9]. Another key role of IL-6 is the stimulation of prostaglandin E2 (PGE2) secretion, as IL-6 deficiency has been shown to reduce PGE2 levels and impair the suppression of inflammatory responses [9]. Interestingly, IL-6 also exerts intracellular regulatory functions, as genetic suppression of IL-6 leads to inhibition of T cell proliferation [10].

### 2.2. IL-10

In contrast to IL-6, IL-10 primarily acts as a potent anti-inflammatory cytokine and plays a central role in MSC-mediated immunosuppression. IL-10 is produced by multiple immune cells, including macrophages, dendritic cells, B cells, and regulatory T cells (Tregs) [11]. Following MSC administration, IL-10 levels increase as part of a counterregulatory response to inflammation; however, its production is largely dependent on the inflammatory context [11].

Through IL-10 secretion, MSCs suppress dendritic cell maturation, leading to the development of an immunosuppressive phenotype. Notably, dendritic cells exposed to both TNF-α and MSC-derived IL-10 fail to upregulate maturation markers and are unable to activate effector T cells [12]. Furthermore, IL-10 plays a critical role in mediating the effects of the MSC secretome, as suppression of T cell proliferation by MSC-conditioned media occurs via IL-10-dependent paracrine signaling without requiring direct cell-to-cell contact [13]. Additionally, IL-10 downregulates MHC class II expression on macrophages and inhibits pro-inflammatory cytokine production in Th1 cells [14].

### 2.3. TGF-β

Together with IL-10, TGF-β represents a key anti-inflammatory mediator that contributes to the immunosuppressive activity of MSCs. TGF-β is a central regulator of immune tolerance and is directly involved in the suppression of CD8+ T cell activity. Elevated levels of TGF-β1 have been observed in co-culture systems of bone marrow-derived MSCs and CD8+ T cells, and neutralization of TGF-β partially restores T cell proliferation, confirming its inhibitory role [15].

Beyond its direct effects on cytotoxic T cells, TGF-β modulates broader immune responses by reducing IFN-γ production and promoting a shift toward anti-inflammatory immune phenotypes [16]. For instance, TGF-β-engineered MSCs have demonstrated therapeutic efficacy in a type 1 diabetes model by shifting the immune response from Th1 to Th2 dominance, resulting in improved insulin levels and survival [17]. Moreover, TGF-β plays a critical role in MSC-mediated expansion of regulatory T cells (CD4+CD25+FoxP3+), further reinforcing immune tolerance [18,19].

### 2.4. TNF-α

While IL-10 and TGF-β mediate anti-inflammatory effects, TNF-α serves as a key pro-inflammatory signal that activates and “licenses” MSC immunomodulatory function. In resting conditions, MSCs exhibit limited immunoregulatory activity; however, exposure to TNF-α triggers activation of the NF-κB signaling pathway via TNFR1, which is essential for their immunosuppressive capacity [20].

TNF-α enhances MSC interaction with immune cells by upregulating adhesion molecules such as ICAM-1, thereby facilitating direct inhibition of T cell activation [21]. In addition, TNF-α increases MSC sensitivity to IFN-γ by upregulating IFN-γ receptor expression, leading to activation of downstream pathways, including STAT5 and p38-MAPK. This results in enhanced secretion of immunosuppressive factors such as IDO and PD-L1/PD-L2 [22].

Importantly, TNF-α stimulation induces a shift in MSC phenotype toward an anti-inflammatory state characterized by increased production of IL-10 and TGF-β, highlighting its role as a key initiator of the MSC-mediated regulatory cascade [23,24].

### 2.5. IL-1β

Similar to TNF-α, IL-1β functions as a pro-inflammatory licensing signal that enhances MSC immunomodulatory activity. Produced primarily by activated macrophages, IL-1β activates NF-κB signaling in MSCs and amplifies downstream immunosuppressive responses [22,25].

IL-1β stimulation induces the secretion of granulocyte colony-stimulating factor (G-CSF) and promotes the production of anti-inflammatory mediators, including IL-10, while reducing pro-inflammatory cytokine levels in target cells such as microglia [26]. In response, MSCs also upregulate IL-1 receptor antagonist (IL-1Ra), which acts as a critical negative regulator of IL-1 signaling.

IL-1Ra plays a central role in mediating the anti-inflammatory effects of MSCs by suppressing macrophage activation, reducing dendritic cell antigen presentation, and promoting regulatory T cell expansion [27]. The importance of this pathway is underscored by studies showing that IL-1Ra-deficient MSCs fail to control inflammation and cannot effectively modulate the Th17/Treg balance [28].

Collectively, these cytokines function as an integrated regulatory network that enables MSCs to sense inflammatory signals and dynamically adapt their immunomodulatory responses through coordinated feedback mechanisms. Within this network, pro-inflammatory cytokines such as TNF-α and IL-1β act as licensing signals that initiate MSC activation. At the molecular level, MSC-mediated cytokine regulation is largely governed by key signaling pathways such as NF-κB and JAK/STAT. Pro-inflammatory cytokines, including TNF-α and IL-1β, activate NF-κB signaling, which in turn induces the expression of immunosuppressive mediators. Similarly, JAK/STAT signaling contributes to the amplification and modulation of cytokine responses, allowing MSCs to dynamically adapt to inflammatory environments.

### 2.6. Cytokine Synergy and Feedback Loops

The cytokines described above do not act in isolation; their combined effects are governed by synergistic activation and feedback regulation. Combined exposure to TNF-α and IFN-γ produces substantially greater upregulation of IDO, PD-L1, and PD-L2 in MSCs than either cytokine alone, indicating non-redundant convergence of NF-κB and JAK/STAT pathways on shared immunoregulatory output. IL-1β and TNF-α similarly co-amplify NF-κB signaling and jointly drive secretion of G-CSF, IL-1Ra, and IL-6, sensitizing MSCs to subsequent IFN-γ stimulation.

On the effector side, IL-10 and TGF-β reinforce one another through positive feedback: MSC-induced regulatory T cells (Tregs) themselves secrete IL-10 and TGF-β, sustaining and propagating the suppressive tone established by the MSCs. The same effector cytokines simultaneously generate negative feedback on the licensing inputs—IL-10-driven M1→M2 macrophage polarization reduces local TNF-α and IL-1β production, while TGF-β suppresses IFN-γ output from effector T cells. The result is a self-stabilizing network in which inflammatory licensing produces an immunosuppressive response that limits its own initiating signal, allowing MSCs to fine-tune the magnitude and duration of immune suppression to the local inflammatory load.

## 3. Diseases

Diseases discussed in this section are grouped according to their underlying immunopathological mechanisms to better highlight differences in MSC-mediated immunoregulation.

### 3.1. Autoimmune Diseases

#### 3.1.1. Systemic Lupus Erythematosus (SLE)

Systemic lupus erythematosus (SLE) is a chronic autoimmune disease characterized by loss of immune tolerance, production of autoantibodies, and persistent systemic inflammation leading to multi-organ damage. The disease is associated with increased mortality, particularly due to cardiovascular complications, infections, and renal involvement [29,30].

The pathogenesis of SLE is driven by dysregulated interactions between innate and adaptive immune responses, including abnormal activation of B and T lymphocytes, increased production of pro-inflammatory cytokines, and enhanced interferon signaling. These processes contribute to sustained immune activation and tissue injury.

In the context of MSC therapy, SLE represents a relevant model due to its pronounced immune imbalance and chronic inflammatory state. MSCs exert immunomodulatory effects through both direct cell–cell interactions and paracrine signaling, involving the secretion of regulatory mediators such as IL-10, TGF-β, prostaglandin E2 (PGE2), and indoleamine 2,3-dioxygenase (IDO) [24,31,32,33,34,35].

Functionally, MSCs suppress the activation and proliferation of T lymphocytes and inhibit the differentiation of pro-inflammatory subsets, including Th1, Th17, and T follicular helper (Tfh) cells. At the same time, MSCs promote the expansion of regulatory T cells (Tregs) and enhance IL-10 production, contributing to the restoration of immune tolerance [36].

In addition, MSCs modulate dendritic cell function by promoting the formation of regulatory phenotypes (DCregs), reducing antigen presentation, and suppressing pro-inflammatory cytokine production. These effects collectively contribute to attenuation of immune activation and reduction in tissue damage.

Clinical studies further support the therapeutic potential of MSCs in SLE. Treatment with umbilical cord-derived MSCs in patients with refractory disease has been associated with increased Treg levels, elevated TGF-β, and decreased Th17 cells and pro-inflammatory cytokines such as IL-17 and TNF-α [37].

Despite these promising findings, the precise mechanisms underlying MSC-mediated immunoregulation in SLE remain incompletely understood, highlighting the need for further investigation.

#### 3.1.2. Systemic Sclerosis

Systemic sclerosis (SSc) is a chronic autoimmune connective tissue disease characterized by vascular dysfunction, immune dysregulation, and progressive fibrosis of the skin and internal organs. The disease is associated with significant morbidity and mortality, and current immunosuppressive therapies, such as mycophenolate, remain insufficient and are often accompanied by adverse effects [38,39].

Due to its combined inflammatory and fibrotic nature, systemic sclerosis represents a relevant target for MSC-based therapy. MSCs exert therapeutic effects primarily through immunomodulation, inhibition of fibrotic pathways, and secretion of paracrine factors.

Mechanistically, MSCs regulate immune responses by increasing anti-inflammatory cytokines such as IL-10 and promoting the generation of regulatory immune cells, including regulatory B cells (Bregs) [40]. In addition, MSCs inhibit pro-fibrotic signaling pathways, particularly TGF-β1/Smad3, thereby reducing collagen synthesis and tissue fibrosis. MSC-derived extracellular vesicles (EVs), including miRNA-containing exosomes, further contribute to anti-fibrotic and immunoregulatory effects [41,42].

MSCs also modulate macrophage activity by reducing CCL2 levels and limiting the recruitment of CCR2-high inflammatory macrophages, thereby attenuating chronic inflammation [43]. Through these combined mechanisms, MSCs influence both immune dysregulation and fibrotic progression in systemic sclerosis.

Clinical evidence suggests modest but measurable therapeutic benefits. Treatment with MSCs has been associated with increased levels of regulatory B cells and IL-10 expression, reduction in skin thickness as measured by the modified Rodnan skin score (mRSS), and improvement in pulmonary arterial pressure and inflammatory markers [40,44].

Despite these promising findings, clinical efficacy remains limited, likely due to the advanced fibrotic stage of the disease at the time of treatment, highlighting the need for earlier intervention and further optimization of MSC-based therapies.

### 3.2. Neurological Disorders

#### 3.2.1. Spinal Cord Injury

Spinal cord injury (SCI) is a severe neurological condition characterized by loss of motor and sensory function due to damage to neural tissue. Current treatments are primarily supportive and focus on stabilization and rehabilitation, with limited capacity to restore neural connectivity [45].

Due to the combination of inflammation, neuronal damage, and limited regenerative capacity, SCI represents a promising target for MSC-based therapy. MSCs exert therapeutic effects through multiple mechanisms, including immunomodulation, neuroprotection, and tissue regeneration.

From an immunological perspective, MSCs modulate the inflammatory microenvironment by promoting the polarization of macrophages from the pro-inflammatory M1 phenotype to the anti-inflammatory M2 phenotype, thereby reducing secondary tissue damage and supporting repair processes [46,47].

In addition, MSCs provide neuroprotective support through the secretion of trophic factors such as nerve growth factor (NGF) and brain-derived neurotrophic factor (BDNF), which enhance neuronal survival, synaptic plasticity, and functional recovery [48,49]. MSCs also contribute to the stabilization of the blood–spinal cord barrier and reduction in oxidative stress, further limiting injury progression.

From a regenerative perspective, MSCs promote axonal growth, remyelination, and the activation of endogenous neural progenitor cells, thereby improving the local microenvironment for neural repair [47].

Preclinical and meta-analytical studies support these mechanisms, demonstrating improved locomotor function following MSC transplantation in experimental models of SCI. Additionally, MSC-derived extracellular vesicles (EVs) have emerged as a promising cell-free therapeutic alternative, providing comparable immunomodulatory and neuroprotective effects with improved stability and safety profiles [50].

Despite these encouraging findings, complete functional recovery remains limited, highlighting the need for further optimization of MSC-based therapeutic strategies.

#### 3.2.2. Ischemic Stroke

Ischemic stroke (IS) is a leading cause of mortality and long-term disability worldwide, accounting for the majority of stroke cases. It results from interruption of cerebral blood flow, leading to oxygen deprivation, neuronal injury, and tissue damage [51,52,53].

The pathophysiology of ischemic stroke involves a complex cascade of events, including neuroinflammation, oxidative stress, excitotoxicity, and apoptosis. These processes contribute to both immediate neuronal death in the ischemic core and progressive damage in the surrounding penumbra, which represents a key target for therapeutic intervention.

Due to the combined presence of inflammation, oxidative stress, and limited regenerative capacity, ischemic stroke represents a promising target for MSC-based therapy. MSCs exert their therapeutic effects primarily through paracrine signaling, rather than direct cell replacement.

Mechanistically, MSCs modulate the post-ischemic inflammatory response by reducing the production of pro-inflammatory cytokines and promoting polarization of microglia toward the anti-inflammatory M2 phenotype, thereby limiting secondary neuronal damage and stabilizing the blood–brain barrier [54].

In addition, MSCs provide neuroprotective effects through the secretion of trophic factors such as vascular endothelial growth factor (VEGF) and brain-derived neurotrophic factor (BDNF), which enhance neuronal survival, promote angiogenesis, and support synaptic plasticity [55]. MSCs also reduce oxidative stress and have been shown to transfer mitochondria to damaged cells, thereby improving cellular metabolism under hypoxic conditions [54].

From a regenerative perspective, MSCs contribute to axonal growth, neurogenesis, and functional recovery by improving the microenvironment within the ischemic brain.

Preclinical and clinical studies suggest that MSC therapy may improve functional outcomes after stroke; however, several limitations remain, including poor cell survival, limited homing efficiency, and variability in therapeutic responses. To address these challenges, advanced strategies such as MSC preconditioning, genetic modification, and the use of MSC-derived extracellular vesicles are currently being investigated [54,55,56,57,58].

Overall, MSC-based therapy represents a multifactorial approach that integrates immunomodulation, neuroprotection, and tissue repair and continues to be a promising direction for the treatment of ischemic stroke.

#### 3.2.3. Diabetes Mellitus

Diabetes mellitus (DM) is a chronic metabolic disorder characterized by persistent hyperglycemia resulting from impaired insulin secretion, insulin resistance, or both. In addition to metabolic dysregulation, DM is increasingly recognized as a condition associated with chronic low-grade inflammation and immune system involvement [59].

Due to its combined metabolic and inflammatory nature, DM represents a relevant target for MSC-based therapy. MSCs exert immunomodulatory and regenerative effects that may address both immune dysfunction and tissue damage, particularly in pancreatic β-cells [60].

In type 1 diabetes mellitus (T1DM), MSCs suppress autoimmune responses by inhibiting T cell activation and reducing pro-inflammatory cytokine production, thereby limiting immune-mediated destruction of β-cells. At the same time, MSCs promote regulatory immune cell populations and enhance anti-inflammatory cytokine signaling, contributing to the restoration of immune tolerance [61].

Mechanistically, MSCs protect pancreatic β-cells in T1DM through TGF-β-driven Th1→Th2 polarization, IDO- and PGE2-mediated suppression of autoreactive CD8+ T cells, and expansion of FoxP3+ regulatory T cells that limit β-cell destruction. TGF-β-engineered MSCs have been shown in preclinical T1DM models to prolong β-cell survival, restore insulin secretion, and improve glycemic control beyond what is achieved with unmodified MSCs.

In type 2 diabetes mellitus (T2DM), MSCs improve metabolic function by reducing chronic inflammation and enhancing insulin sensitivity. MSC-derived factors modulate cytokine profiles, decreasing levels of pro-inflammatory mediators such as TNF-α and IL-6, which are known to contribute to insulin resistance [62,63].

In gestational diabetes mellitus (GDM), MSCs exert immunoregulatory effects at the maternal-placental interface. By reducing local TNF-α and IL-6 levels and modulating macrophage polarization in adipose and placental tissue, MSCs may restore the maternal-fetal cytokine balance that is disrupted in GDM. Preclinical evidence further indicates that MSC-derived extracellular vesicles can attenuate placental inflammation and improve insulin sensitivity, though clinical translation remains preliminary.

In addition to immunomodulation, MSCs support tissue regeneration through paracrine mechanisms, including the secretion of growth factors and extracellular vesicles that promote β-cell survival, angiogenesis, and repair of damaged tissues [64].

Preclinical and early clinical studies suggest that MSC therapy may improve glycemic control and reduce inflammatory markers in patients with diabetes. However, variability in treatment protocols, limited cell survival, and incomplete restoration of β-cell function remain important challenges [65,66].

Overall, MSC-based therapy represents a promising approach that integrates immunoregulation and metabolic support, although further studies are required to optimize its clinical efficacy.

#### 3.2.4. Female Infertility

Female infertility is often associated with ovarian dysfunction, including conditions such as premature ovarian failure (POF) and primary ovarian insufficiency (POI), which are characterized by impaired folliculogenesis, hormonal imbalance, and reduced ovarian reserve [67,68,69]. In addition to endocrine disruption, inflammatory processes and cellular apoptosis contribute to the deterioration of the ovarian microenvironment.

Due to their regenerative and immunomodulatory properties, MSCs have emerged as a promising therapeutic approach for restoring ovarian function. MSCs exert their effects primarily through paracrine signaling, including the secretion of growth factors such as insulin-like growth factor-1 (IGF-1), vascular endothelial growth factor (VEGF), and hepatocyte growth factor (HGF), which support cell survival, angiogenesis, and tissue regeneration [70,71].

Mechanistically, MSCs reduce apoptosis of granulosa cells, enhance follicular development, and promote restoration of hormonal balance. These effects are associated with increased levels of estradiol (E2) and anti-Müllerian hormone (AMH), along with decreased levels of follicle-stimulating hormone (FSH), reflecting improved ovarian function [72].

MSCs also modulate inflammatory pathways by reducing pro-inflammatory cytokines such as TNF-α, IL-6, and IFN-γ, thereby restoring the ovarian microenvironment and supporting tissue repair. In autoimmune-related ovarian dysfunction, MSCs help suppress immune-mediated damage and promote regeneration of ovarian tissue [73].

Additionally, MSC-derived extracellular vesicles, particularly exosomes containing regulatory microRNAs, play a key role in mediating therapeutic effects. These vesicles modulate signaling pathways such as PI3K/AKT, inhibit apoptosis via suppression of caspase-3 activity, and enhance vascularization through upregulation of VEGF [74].

Preclinical studies demonstrate that MSC therapy can improve ovarian morphology, increase follicle number, and restore reproductive function. However, clinical translation remains limited, and further studies are required to optimize therapeutic protocols and evaluate long-term efficacy.

## 4. Discussion

Mesenchymal stromal cells (MSCs) demonstrate therapeutic potential across a wide range of diseases despite substantial differences in their pathophysiology. A common feature shared among systemic sclerosis, female infertility, spinal cord injury, systemic lupus erythematosus (SLE), ischemic stroke (IS), and diabetes mellitus is the presence of chronic inflammation, immune dysregulation, cellular damage, and impaired regenerative capacity. MSCs primarily target these pathological processes through immunomodulation, anti-inflammatory signaling, and paracrine activity mediated by their secretome.

In autoimmune diseases such as SLE, MSC therapy demonstrates significant therapeutic potential due to its capacity to regulate dysregulated immune responses. MSCs suppress activation and differentiation of pro-inflammatory T cell subsets, including Th1, Th17, and T follicular helper (Tfh) cells, while promoting expansion of regulatory T cells (Tregs), thereby contributing to restoration of immune homeostasis. Furthermore, MSCs decrease production of pro-inflammatory cytokines such as IL-17 and TNF-α and increase anti-inflammatory mediators, including IL-10 and TGF-β, resulting in reduced inflammation, improved immune tolerance, and attenuation of tissue damage. Collectively, these mechanisms suggest that MSC therapy may partially re-establish immune balance in SLE patients.

Similarly, MSC therapy shows promising effects in systemic sclerosis, where disease progression is strongly associated with immune dysregulation and progressive fibrosis. Clinical improvements, including reduced skin thickness and decreased inflammatory markers, have been reported following MSC treatment. However, the overall therapeutic effect remains limited, likely due to irreversible fibrotic changes and structural tissue remodeling in advanced disease stages, highlighting the importance of early therapeutic intervention.

In neurological disorders such as ischemic stroke and spinal cord injury, MSCs exert therapeutic effects primarily through neuroprotective and regenerative mechanisms. MSCs demonstrate the ability to migrate toward injured tissues, including the ischemic core and penumbra, where they secrete trophic factors such as vascular endothelial growth factor (VEGF), brain-derived neurotrophic factor (BDNF), and nerve growth factor (NGF). These factors promote neurogenesis, angiogenesis, synaptic plasticity, and neuronal survival while reducing apoptosis and oxidative stress. MSCs also modulate neuroinflammation by suppressing pro-inflammatory cytokine production and promoting polarization of immune cells toward anti-inflammatory phenotypes. Additionally, emerging evidence suggests that MSCs may improve cellular bioenergetics through mitochondrial transfer to damaged cells, thereby enhancing tissue repair and functional recovery. Nevertheless, complete neural regeneration remains limited due to intrinsic inhibitory mechanisms of the central nervous system.

In female infertility, MSCs demonstrate strong regenerative potential, particularly in conditions such as premature ovarian failure (POF) and primary ovarian insufficiency (POI). MSC-derived secretome, including growth factors such as IGF-1, VEGF, and hepatocyte growth factor (HGF), as well as exosomal microRNAs, promotes follicular survival, reduces granulosa cell apoptosis, stimulates angiogenesis, and contributes to restoration of hormonal balance. These effects support partial recovery of ovarian function and improve follicular development, although genetic causes of infertility remain less responsive to MSC therapy.

Although currently less extensively studied, MSC therapy also shows potential in diabetes mellitus due to its anti-inflammatory and regenerative properties. In type 1 diabetes, MSCs may suppress autoimmune-mediated destruction of pancreatic β-cells through modulation of immune responses and reduction in inflammatory cytokine production. In type 2 diabetes, MSC therapy may improve insulin sensitivity and reduce chronic low-grade inflammation associated with metabolic dysfunction. Additionally, MSC-derived paracrine factors may contribute to the protection and functional recovery of pancreatic β-cells, potentially improving glucose homeostasis.

Across all discussed diseases, the therapeutic effects of MSCs appear to be mediated predominantly through paracrine mechanisms rather than direct differentiation into tissue-specific cells, highlighting the central role of the MSC secretome in therapeutic efficacy. The MSC secretome, including cytokines, growth factors, extracellular vesicles, and microRNAs, plays a central role in regulating immune responses, suppressing inflammation, and promoting tissue repair. However, several limitations remain, including heterogeneity of MSC sources, variability in treatment protocols, limited cell survival following transplantation, and incomplete restoration of damaged tissues. These challenges highlight the need for further optimization of MSC-based therapies, including standardization of cell preparation methods, identification of optimal dosing strategies, and development of cell-free therapeutic approaches based on MSC-derived extracellular vesicles.

In recent years, MSC-based therapies have progressed from preclinical research to early-phase clinical trials across multiple disease areas, including autoimmune disorders, neurological conditions, and metabolic diseases. These studies have consistently demonstrated that MSC therapy is generally safe and well tolerated; however, clinical efficacy remains variable.

Several challenges continue to limit successful clinical translation, including heterogeneity of MSC sources, differences in manufacturing protocols, suboptimal cell survival after transplantation, and limited homing efficiency to target tissues. To address these limitations, emerging strategies have focused on enhancing MSC functionality through genetic engineering, preconditioning approaches, and optimization of delivery methods.

In particular, MSC-derived extracellular vesicles (EVs), including exosomes, have attracted significant attention as a promising cell-free therapeutic alternative. These vesicles retain key immunomodulatory and regenerative properties of MSCs while offering advantages in terms of safety, scalability, and standardization.

Overall, MSC therapy represents a promising and versatile therapeutic strategy capable of addressing complex immune-mediated and degenerative diseases through coordinated immunomodulatory, anti-inflammatory, and regenerative mechanisms. While current evidence supports the safety and potential efficacy of MSC-based interventions, further experimental and clinical studies are required to improve therapeutic consistency and to better define the long-term clinical benefits of MSC therapy.

## 5. Conclusions

Mesenchymal stromal cells (MSCs) represent a promising therapeutic tool due to their combined regenerative and immunomodulatory properties. The evidence discussed in this review demonstrates that MSC-mediated immunoregulation is primarily governed by a coordinated cytokine network involving IL-6, IL-10, TGF-β, TNF-α, and IL-1β, which collectively modulate both innate and adaptive immune responses. Through interactions with dendritic cells, macrophages, T lymphocytes, and other immune cell populations, MSCs promote a shift from pro-inflammatory to tolerogenic immune phenotypes, supporting tissue repair and limiting chronic inflammation.

Across multiple disease contexts—including systemic lupus erythematosus, systemic sclerosis, ischemic stroke, spinal cord injury, diabetes mellitus, and female infertility—MSC therapy demonstrates consistent mechanisms of action centered on cytokine-mediated suppression of excessive immune activation and restoration of immune homeostasis. Importantly, the therapeutic effects of MSCs appear to rely predominantly on paracrine signaling via soluble factors and extracellular vesicles rather than direct differentiation into tissue-specific cell types, highlighting the significance of the MSC secretome as a key mediator of clinical benefit.

Despite substantial progress, several challenges remain, including heterogeneity of MSC populations, limited survival following transplantation, and the need for standardized protocols regarding cell source, dose, and route of administration. Addressing these limitations will be essential for improving reproducibility and maximizing therapeutic efficacy in clinical applications.

Overall, MSC-based therapy represents a multifactorial and adaptable approach capable of targeting complex immune-mediated pathologies through cytokine-driven mechanisms. Continued investigation into MSC biology, cytokine signaling pathways, and optimization of therapeutic strategies will be critical for translating experimental findings into safe and effective clinical treatments.

## Figures and Tables

**Table 1:** MSC-relevant cytokine network: licensing inputs, effector mediators, and context-dependent factors.

Cytokine	Source	Receptor	Pathway	Role	Net Effect on MSC Immunomodulation
TNF-α	Activated macrophages, T cells	TNFR1	NF-κB	Licensing	Upregulates ICAM-1, IFN-γR, IDO, PD-L1; primes suppressive phenotype
IL-1β	Macrophages (inflammasome)	IL-1R1	NF-κB	Licensing	Drives G-CSF and IL-1Ra; sensitizes MSCs to IFN-γ signaling
IFN-γ	Activated T cells, NK cells	IFN-γR1/2	JAK/STAT1	Licensing	Induces IDO, PD-L1, MHC-II; required for full suppression
IL-10	MSCs, macrophages, Tregs	IL-10R	JAK/STAT3	Effector	M1→M2 macrophage polarization; T cell suppression
TGF-β	MSCs, Tregs	TβRI/II	SMAD2/3	Effector	CD8+ T cell suppression; FoxP3+ Treg expansion
IL-6	MSCs, many cell types	IL-6R/gp130	JAK/STAT3	Context-dependent	DC inhibition; PGE2 induction; intracellular regulation of T cell proliferation

## Data Availability

No new data were created or analyzed in this study.
